# Inflammatory stimulation preserves physiological properties of retinal ganglion cells after optic nerve injury

**DOI:** 10.3389/fncel.2014.00038

**Published:** 2014-02-12

**Authors:** Henrike Stutzki, Christian Leibig, Anastasia Andreadaki, Dietmar Fischer, Günther Zeck

**Affiliations:** ^1^Neurochip Research Group, Natural and Medical Sciences Institute, University of TübingenReutlingen, Germany; ^2^Graduate Training Centre of NeuroscienceTübingen, Germany; ^3^Department of Neurology, Experimental Neurology, Heinrich Heine University DüsseldorfDüsseldorf, Germany

**Keywords:** retinal ganglion cells, optic nerve injuries, neuroregeneration, microelectrode array, inflammatory stimulation, axonal imaging

## Abstract

Axonal injury in the optic nerve is associated with retinal ganglion cell (RGC) degeneration and irreversible loss of vision. However, inflammatory stimulation (IS) by intravitreal injection of Pam_3_Cys transforms RGCs into an active regenerative state enabling these neurons to survive injury and to regenerate axons into the injured optic nerve. Although morphological changes have been well studied, the functional correlates of RGCs transformed either into a de- or regenerating state at a sub-cellular level remain unclear. In the current study, we investigated the signal propagation in single intraretinal axons as well as characteristic activity features of RGCs in a naive, a degenerative or a regenerative state in *ex vivo* retinae 1 week after either optic nerve cut alone (ONC) or additional IS (ONC + IS). Recordings of single RGCs using high-density microelectrode arrays demonstrate that the mean intraretinal axonal conduction velocity significantly decreased within the first week after ONC. In contrast, when ONC was accompanied by regenerative Pam_3_Cys treatment the mean intraretinal velocity was undistinguishable from control RGCs, indicating a protective effect on the proximal axon. Spontaneous RGC activity decreased for the two most numerous RGC types (ON- and OFF-sustained cells) within one post-operative week, but did not significantly increase in RGCs after IS. The analysis of light-induced activity revealed that RGCs in ONC animals respond on average later and with fewer spikes than control RGCs. IS significantly improved the responsiveness of the two studied RGC types. These results show that the transformation into a regenerative state by IS preserves, at least transiently, the physiological functional properties of injured RGCs.

## Introduction

Retinal ganglion cells (RGCs) convey visual signals from the retina along their axons through the optic nerve to the brain. Axonal injuries in the optic nerve normally result in permanent functional loss due to regenerative failure of RGCs.

In animal models cut or crush of the optic nerve mimics the damage that occurs in traumatic optic neuropathy and the cell death of RGCs observed in glaucoma (Goldblum and Mittag, 2002; Diekmann and Fischer, 2013). Diseases like glaucoma or mechanical stress impair axonal transport, lead to synapse retraction and dying back degeneration of the axon (Raff et al., 2002). As a consequence cell soma and dendrites may shrink and apoptosis is initiated (Jakobs et al., 2005; Whitmore et al., 2005; Diekmann and Fischer, 2013). In rodents RGCs start to undergo apoptotic cell death 1 week after intraorbital optic nerve injury (Berkelaar et al., 1994; Fischer et al., 2004; Fischer and Leibinger, 2012; Germain et al., 2013) Degeneration of the different cellular compartments (axon, soma, dendrites) might not occur simultaneously but rather independently and compartmentalized. Neurons can initiate apoptosis specifically for each of their compartments: axon, soma and dendrites (Raff et al., 2002).

The health-condition of RGCs is reflected by their functionality. A variety of studies examined RGC functionality in injured retinae using visual evoked potential recordings and electro-retinograms that quantify population responses in the visual cortex or the retina. These studies revealed a decrease in signal amplitudes (Maffei et al., 1985; Heiduschka et al., 2010) changes in average response latencies (Bain et al., 2001) and loss of spatial resolution (Kloecker et al., 2001). However, the results of these methods are based on average signals measured outside the eye. It remains unclear whether the reported changes are a consequence of decreased cell density or arise at single-cell level which would be necessary to investigate the health status of surviving RGCs or susceptibility to regenerative treatments after optic nerve cut. Only a few studies investigated individual neurons in injured mammalian retinae (Takao et al., 2002; Weber and Harman, 2005; Feng et al., 2013) but never in growth-stimulated RGCs.

Several studies have demonstrated that mature RGCs can be transformed into a regenerative state by inflammatory stimulation (IS) enabling these neurons to survive optic nerve injury and to regenerate lengthy axons into the injured optic nerve (Fischer et al., 2000; Fischer, 2012). IS can be induced by lens injury or intravitreal application of toll-like receptor 2 agonists such as Pam_3_Cys (Fischer et al., 2001; Hauk et al., 2010), which induce the expression and release of neuroprotective and axon growth-promoting cytokines such as CNTF, LIF and IL-6 from retinal astrocytes and Müller cells (Muller et al., 2007; Leibinger et al., 2009, 2013). Although the neuroprotective and axon growth promoting effects of IS have been morphologically characterized the effect on the health condition of surviving RGC remained unknown.

Therefore, the present study investigated the functional changes occurring in RGCs following ONC alone and in regenerative RGCs obtained by additional IS. We used two types of microelectrode arrays: a high-density metal-based microelectrode array (MEA) (Feng et al., 2013) and a CMOS-based high-density transistor array with sensor spacing of 7.4 μm (Neurochip) (Lambacher et al., 2011). The high spatial resolution of the latter array enables the recording of RGC populations at the single somatic as well as axonal level (Menzler and Zeck, 2011; Zeck et al., 2011). We report that a regenerative treatment of injured RGCs protects their physiological functionality on the axonal as well as on the somatic level.

## Materials and methods

### Surgical procedure

Surgical procedures were approved by the local authorities (LanuV, Recklinghausen, Germany) and adhered to the ARVO Statement for the Use of Animals in Ophthalmic and Vision Research. In total 18 Sprague-Dawley rats (22–23 days old) were used, 6 for each experimental group (ONC, ONC + IS) and 6 for the control group (Ctr). In the experimental group, rats were anesthetized by intraperitoneal injections of ketamine (60–80 mg/kg) and xylazine (10–15 mg/kg). A 1- to 1.5-cm incision was made in the skin above the right orbit. The optic nerve was surgically exposed under an operating microscope, the dural sheath was longitudinally opened, and the nerve was completely cut 1 mm behind the eye by means of jeweller's forceps, avoiding injury to the retinal artery. The vascular integrity of the retina was verified by funduscopic examination after surgery. Rats received intravitreal injection of either 10 μL PBS or 10 μg (S)-[2,3-Bis(palmitoyloxy)-(2-RS)-propyl]-*N*-palmitoyl-(R)-Cys-(S)-Ser-(S)-Lys4-OH (Pam_3_Cys) (EMC Microcolections, Tübingen, Germany) solved in 10 μL PBS. The intravitreal injection was performed simultaneously to optic nerve cut. The integrity of the lens was carefully verified for each animal. Control animals received no surgery and treatment at all.

### Preparation of the retina

One week after surgery (postoperative day 5–7), rats were dark adapted for 30 min, anesthetized by CO_2_ and killed by cervical dislocation under dim red light (640 nm LEDs; Roithner Lasertechnik, Vienna, Austria). Isolated eyes were placed in a petri dish with oxygenated Ames' medium (A 1420, Sigma Aldrich, Germany) under a dissecting microscope illuminated with the dim red light. Eyes were hemisected, the vitreous body was carefully removed and the retina was peeled off the sclera. The retina was dissected into retinal portions, which extended well beyond the recording area of the electrode array. Retinal portions were mounted ganglion cell side down on the electrode array and were used for recordings immediately. The array surface was coated previously with poly-L-lysine hydrobromide (1 mg/ml in ultra-pure water, 150 kDa molecular weight; Sigma Aldrich, Germany). During recordings, retinae were constantly perfused with warm (35–37°C) oxygenated Ames' medium (A 1420, Sigma Aldrich, Germany) at a rate of 5–7 ml/min.

### Recording

Recordings were performed with two different types of microelectrode arrays—a Neurochip and an array comprising 252 metal-based electrode (100 μm electrode spacing; Multichannelsystem MCS GmbH). The Neurochip is a CMOS-based, high-density transistor array, which allows recordings on a subcellular level (Lambacher et al., 2011; Menzler and Zeck, 2011; Zeck et al., 2011). It comprises an array of 128 × 128 equally spaced (7.4 μm) sensors covering an area of 1 mm^2^. Because of the high spatial resolution axonal action potentials of individual RGCs can be recorded and the axonal conduction velocity of single intraretinal axons can be calculated. As default configuration every second column (128 × 64 sensors) was measured achieving for each sensor a sampling frequency of 12 kHz. To identify the physiological type of the recorded RGCs, light stimuli were presented during recording. Spike identification and assignment to the corresponding cell was performed as described in previous reports (Zeck and Masland, 2007; Feng et al., 2013).

The maintained and light-induced activity of RGCs shown in this study (Figures 3, 4) was recorded using a 252 MEA, as the continuous recording time of the current Neurochip setup was limited to a few tens of seconds.

Retinae from at least three animals per group were used for recordings on each setup. From each retina 1–3 retinal portions were recorded. Numbers of RGCs were pooled over the recorded retinal portions of one group (ONC, ONC + IS or Ctr). Although the signal waveforms are similar on the two setups, as demonstrated in Zeck et al. (2011), data were not pooled as slight. The reason is that slightly different values for the evaluated parameters are obtained, which is likely attributed to slight technical differences (i.e., restricted continuous recording time of several seconds on the Neurochip or large electrode spacing on the MEA).

### Temperature monitoring

**Figure 1:**
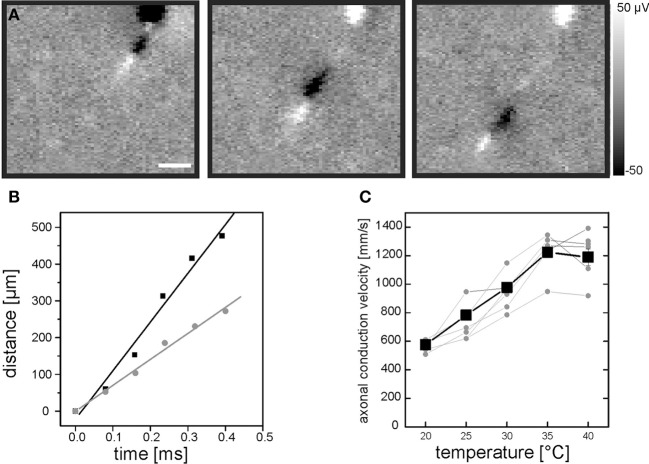
**Evaluation of intraretinal axonal conduction velocity. (A)** A sequence of three electrical images illustrates the propagation of an axonal action potential across the CMOS-based high-density sensor array. The first electrical image is recorded 200 μs after the occurrence of a somatic spike. The interval between the electrical images is 200 μs. Scale bar indicates 150 μm. The gray scale shows calibrated voltages. The cell soma is located at the top of the electrical images. **(B)** The distance between the positive axonal peak [white leading peaks in **(A)**] and the soma location increases linearly over time. The distance-time relation is shown for two recordings of the same neuron at two different temperatures: 35°C (black symbols) and 25°C (gray symbols). **(C)** Axonal conduction velocity increases with temperature. Axonal conduction velocities of 10 RGCs were evaluated in one retina (five of them are shown). Each gray symbol represents the velocity calculated at a given temperature. Velocities for one RGC are connected by lines for visualization purpose. The mean velocity is shown as black line. The axonal conduction velocity increases linearly in the range between 20°C and 35°C.

A chip-integrated resistive sensor allows for continuous monitoring of the electrolyte temperature above the CMOS-based sensor array (Eversmann et al., 2003). A regulation loop maintains a constant user-specified temperature utilizing a peltier element located underneath the sensor chip and a temperature controlled perfusion (MultiChannelsystems MCS GmbH). Exact monitoring of the temperature appears crucial, as it strongly affects the axonal conduction velocity (Figure 1C).

### Identification of somatic signals and data analysis

An offline algorithm was used that allowed to separately identifying somatic and axonal signals (for signal characteristics see Figure 1A). A detailed description of the identification of somatic action potentials and their assignment to the corresponding cell was given previously (Lambacher et al., 2011). In brief, the analysis proceeds in three main steps: (1) all points in spatiotemporal data space are screened for threshold crossings of a signal vector *V* obtained by summing over voltage values of neighbouring sensors, (2) threshold crossings are grouped to single action potentials, and (3) action potentials are assigned to individual neurons. The signal vector *V* for the first step is defined as (Lambacher et al., 2011)

(1) $$V=\sqrt{\sum_{i=1}^{n}{\left(\frac{V_{i}}{\sigma_{i}}\right)}^{2}}$$

with *V*_*i*_ the signal amplitude of data point *i* in the local neighbourhood and σ_*i*_ the root mean square (rms) noise of the respective sensor in the local neighbourhood. The dimensionality of the local neighbourhood containing and surrounding the data point under consideration is given by *n*. For this study an asymmetric neighbourhood was chosen (5 sensor rows, 3 sensor columns and 3 time steps) since only every second column was measured. A threshold value of *V* = 13 turned out to be robust to separate somatic signals from noise. Steps (2) and (3) were unchanged with respect to previous reports (Lambacher et al., 2011). Finally, areas of the sensor grid which contained action potentials from one cell only, were identified in supervised manner.

### Identification of axonal signals

In rat retinae, axonal extracellular signals are rarely detected in the raw recordings but are hidden in the sensor noise. To reveal axonal signals a methodology described by Petrusca et al. (2007) was adapted. The spike triggered average was defined ν_*ij*_(*t*_*k*_) for any sensor (row: *i*; column: *j*) determined by the spike-train *t*_1_, …, *t*_*n*_ of a RGC soma (located at a specific row *i*′ and column *j*′) as

(2) $${\bar{v}}_{ij}\left(t_{k}\right)=\frac{1}{n}\sum_{l=1}^{n}v_{ij}\left(t_{l}+t_{k}\right),\forall i,j,k$$

with: *n* : the total number of spikes in the spike train; *t*_*l*_: time of occurrence of spike l; *v*_*ij*_(*t*_*l*_ + *t*_*k*_): voltage at sensor row *i*, column *j* and time *t*_*l*_ + *t*_*k*_.

By evaluation of expression (2) for all sensors *i* and j at a given timepoint *k*, one obtains a two-dimensional voltage map at the temporal offset *t*_*k*_ to the somatic spike. Negative *k* values allow viewing into the (average) history of the spikes, positive *k* values into the (average) future which may contain an axonal signal. Axonal action potentials become visible because the spatiotemporally uncorrelated noise is suppressed by the averaging procedure. About 30 spikes were used to construct the spike triggered average. An example is given in Figure 1A. Using this approach only axons originating from a recorded cell body are detected. Signals from axons of passage are not identified as no temporal reference signal (somatic spike) is available.

### Evaluation of the intraretinal conduction velocity

For the velocity of a single propagating action potential, spatial locations of its biphasic peak were determined by respective extreme values within each time frame. The axon's path was assumed to be linear (intraretinal axons run nearly parallel) between consecutive time frames. The axon path on the sensor array was constructed by concatenation of linear pieces as shown in Figure 1. The temporal gradients introduced by the readout scheme of the chip across different sensor columns [typical value: (11.5 kHz·64)^−1^ ≈1.4 μs per column] was corrected. Gradients across different sensor rows can be neglected.

### Maintained and light-induced RGC activity

Because the firing rate of RGCs depends on the adaptation level, recordings started after 10 min of adaptation to a constant background illumination. First the maintained (spontaneous) activity was recorded at background illumination. Thereafter full-field flashes of 1 Hz were presented to the same retina for 30 s. Visual stimuli were presented with a LED (Luxeon, 505 nm) focused through the transparent MEA onto the photoreceptor layer of the retina. Periodic flashes were produced by switching the LED every 500 ms between two light levels (ON phase vs. OFF phase) with a contrast of 97%. The stimulus light level was set by a customized circuit driven by a commercial stimulus generator (STG, MCS GmbH) to 800 mW/m^2^. Intensity values were determined at the focus plane of the retina (Optical Meter 1835-C, Newport Spectra-Physics, Darmstadt, Germany). The contrast was calculated as the ratio |*I*_stim_ − *I*_BG_|/(*I*_stim_ + *I*_BG_) (Michelson contrast). Stimulus presentation triggered the recording system and was preceded by an adaptation period as described above. Light response latency was calculated as the mean first spike latency averaged over 30 repetitions. The calculation is done separately for the ON- and the OFF- phase of the stimulus. First spike latency is defined as the time between stimulus onset and the occurrence of the first spike thereafter. Light responses were evaluated if the cell elicits at least one spike per stimulus presentation.

### Cell type classification

Retinal ganglion cell types were broadly classified based on their response to the flash stimuli into ON, OFF and ON–OFF types. The ON and OFF types were subdivide in transient or sustained cells, respectively (Zeck and Masland, 2007). To identify the cell type the average light response per stimulus phase is considered. Only cells which displayed on average at least one spike per stimulus presentation are evaluated. RGCs were identified as sustained or transient by comparing the number of spikes during the last quarter of the stimulus to the number of spikes throughout the stimulus presentation. For a transient cell the number of spikes in the last quarter is smaller than a quarter of the total number of spikes, while it is larger for a sustained RGC.

### Statistics

For group comparisons the Wilcoxon ranksum test was used, in order to keep significance evaluations non-parametric. Two samples (i.e., mean axonal velocity of RGCs of ONC retinae or control retinae) were supposed to display a significant difference when *p* < 0.05, *p* < 0.01, or *p* < 0.001 (^***^). For all physiological parameters (conduction velocity, firing rate, light-induced firing rate, and latency) means + standard errors of the mean are presented.

## Results

### Axonal conduction velocity in retinae following optic nerve injury and regenerative treatment

Action potentials normally propagate along the axon of a RGCs. Due to the high spatial resolution of the Neurochip (7.4 μm) we were able to record propagating action potentials along multiple intraretinal axons and calculate the intraretinal conduction velocity. The trajectory of the action potential appeared in consecutive electrical images of the spike triggered average for each cell (Figure 1A). Based on these electrical images the intraretinal conduction velocity was calculated as described in the Materials and Methods section (Figure 1B). In every retinal portion the axonal conduction velocity could only be calculated for the subset of recorded RGCs extending their proximal axon over at least 500 μm across the array.

As temperature may strongly affect axonal conduction velocity we initially calibrated the temperature dependency in our recording system (Material and Methods, Figure 1C). We found that in the range between 20°C and 35°C each additional degree raised the conduction velocity by 43 mm/s. We therefore maintained the retinae during the recording strictly at 36 ± 1°C.

To assess functional changes in retinal axons after nerve injury and regeneration treatment, respectively, we evaluated the axonal conduction velocities in single intraretinal axons following either ONC alone or ONC + IS. More specific e*x vivo* retinae derived from rats, which either received unilateral ONC or ONC and additional IS were recorded on postoperative days 5–7. Retinae from naive, untreated rats of the same strain and age were used as controls.

**Figure 2:**
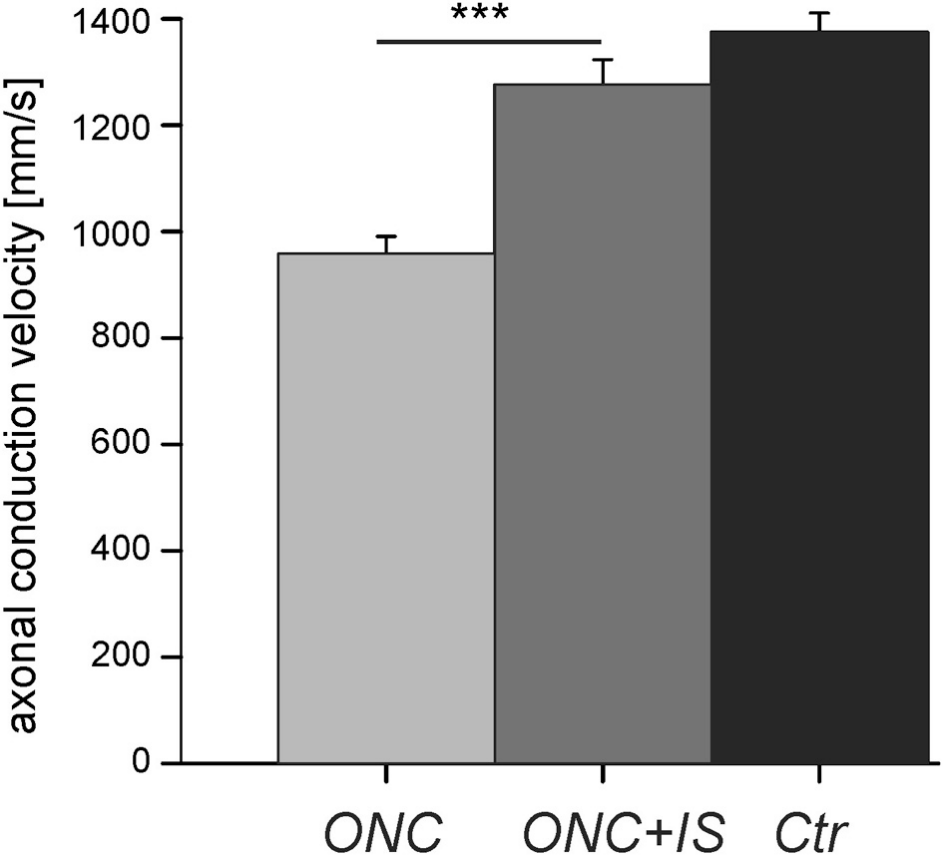
**Reduced axonal conduction velocity after optic nerve cut (ONC) is prevented by additional inflammatory stimulation (ONC + IS)**. Mean intraretinal axonal conduction velocitie of RGCs in ONC retinae (*n* = 16 RGCs), ONC + IS retinae (*n* = 11 RGCs), and control retinae (*n* = 35 RGCs). The mean conduction velocity in ONC retinae is significantly different from the mean of the other two groups (^***^p < 0.001). The mean velocity of control RGCs and RGCs in ONC + IS retinae do not differ significantly.

In control retinae intraretinal axons displayed an average axonal conduction velocity of 1383 ± 37 mm/s (*n* = 36) (Figure 2). Within 1 week (post operative day 5–7) the conduction velocity significantly decreased to 960 ± 33 mm/s (*n* = 16), whereas additional IS prevented the reduction of the axonal conduction velocity. The average intraretinal velocity (1276 ± 47 mm/s, *n* = 11) was significantly higher than in retinae without IS (*p* < 0.001). This value did not significantly differ from the mean intraretinal velocity calculated in control retinae. In all three conditions (control, ONC, and ONC + IS) the conduction velocity in ON and OFF RGCs was similar.

In conclusion, the intraretinal conduction velocity decreases significantly 5–7 days after ONC and was protected by IS.

### Maintained RGC activity

**Figure 3:**
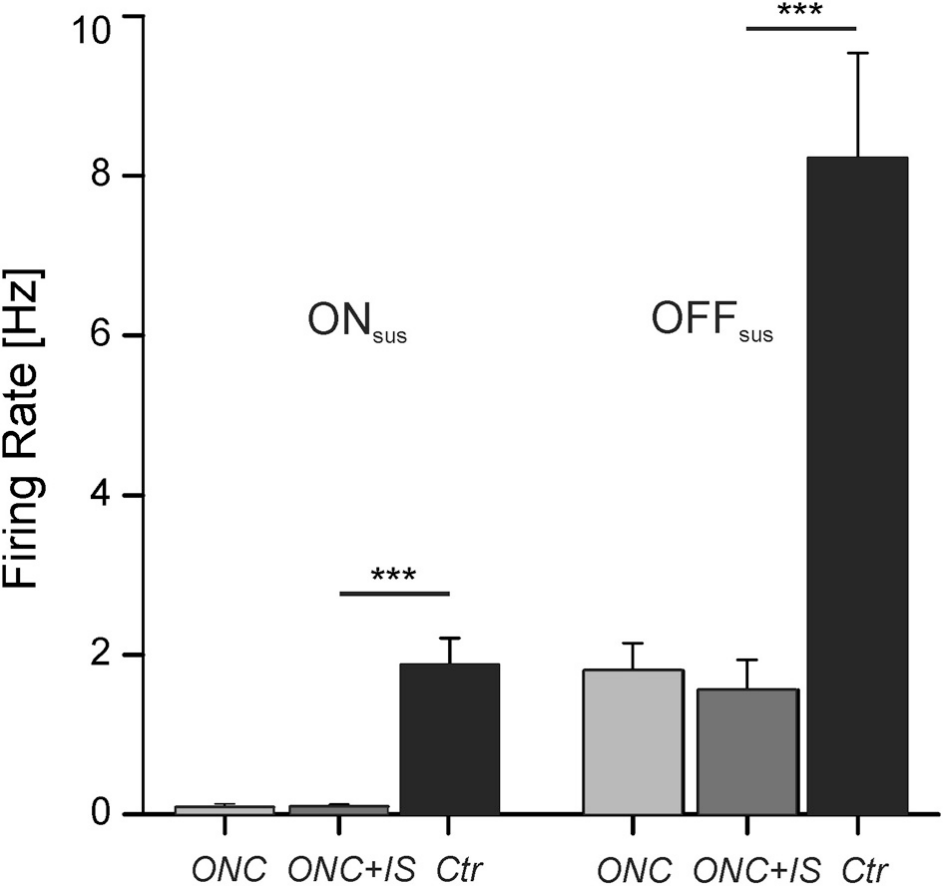
**Maintained RGC activity is decreased by ONC**. Average maintained activity (firing rate) of RGCs after ONC, after ONC + IS and in control condition. On the left side ON sustained RGCs (ON_sus_) are presented, on the right side OFF sustained RGCs (OFF_sus_). After ONC the mean spontaneous firing rate decreases significantly (^***^p < 0.001). Numbers of evaluated RGCs are given in the text.

Axonal injury does not only affect the axon, but also leads to somatic and potentially dendritic degeneration. To investigate the functional changes at the somatic level, we evaluated the maintained RGC activity for the three conditions (ONC, ONC + IS, and control) (Figure 3). Only RGCs which showed a robust light response were evaluated in this experiment.

Maintained or spontaneous activity was defined as stimulus independent RGC spiking and presented as the so-called “firing rate” (action potentials/second). Based on the subsequent light response pattern RGCs were classified as ON, OFF, or ON–OFF type and furthermore as sustained or transient cells (Material and Methods). As transient RGCs showed no maintained activity in our recordings we analyzed sustained RGCs only.

Consistent with previous studies, OFF RGCs exhibited a higher spontaneous activity compared to ON RGCs (Margolis and Detwiler, 2007; Sekirnjak et al., 2011). In healthy control retinae the mean firing rate for OFF_sus_ cells (8.3 ± 1.3 Hz, *n* = 54) was about four times higher than for ON_sus_ cells (1.8 ± 0.3 Hz, *n* = 135). O.K. Noteworthy, in both populations a few number of cells exhibited firing rates deviating from the average firing rate more than fourfold. The maximal firing rate observed in ON_sus_ cells was 25.4 Hz, in OFF_sus_ cells 39.4 Hz. The large variability of the spontaneous activity within one major cell class indicates the existence of further RGC subtypes (Carcieri et al., 2003; Zeck and Masland, 2007; Farrow and Masland, 2011).

Following ONC, the mean spontaneous firing rate significantly decreased for both, ON_sus_ cells [0.1 ± 0.04 Hz (*n* = 43)] and OFF_sus_ cells [1.8 ± 0.3 Hz (*n* = 14)]. The maximal spontaneous firing rate among these cells was 4.8 Hz. IS did not affect the mean spontaneous activity of RGC in ONC retinae irrespective of the cell type. Here the mean spontaneous firing rate was 0.1 ± 0.03 in ON_sus_ cells (*n* = 110) and 1.6 ± 0.4 in OFF_sus_ cells (*n* = 14). The maximal spontaneous firing rate among these RGCs was 5.4 Hz.

In summary, the decreased spontaneous activity of RGCs in ONC retinae was not recovered by IS treatment.

### RGC response to light flashes

Next, we assessed the ability of RGCs to respond to light stimulation, which is an indicator for the functionality of the RGC dendrites and the presynaptic circuitry. A light increment or decrement triggers activity in ON and OFF RGCs, respectively. RGCs respond to a step-wise intensity change with a characteristic latency of ~ 50–100 ms. Changes in the light response, i.e., a delayed response latency, likely indicate a change in the dendritic input and/or dendritic processing.

**Figure 4:**
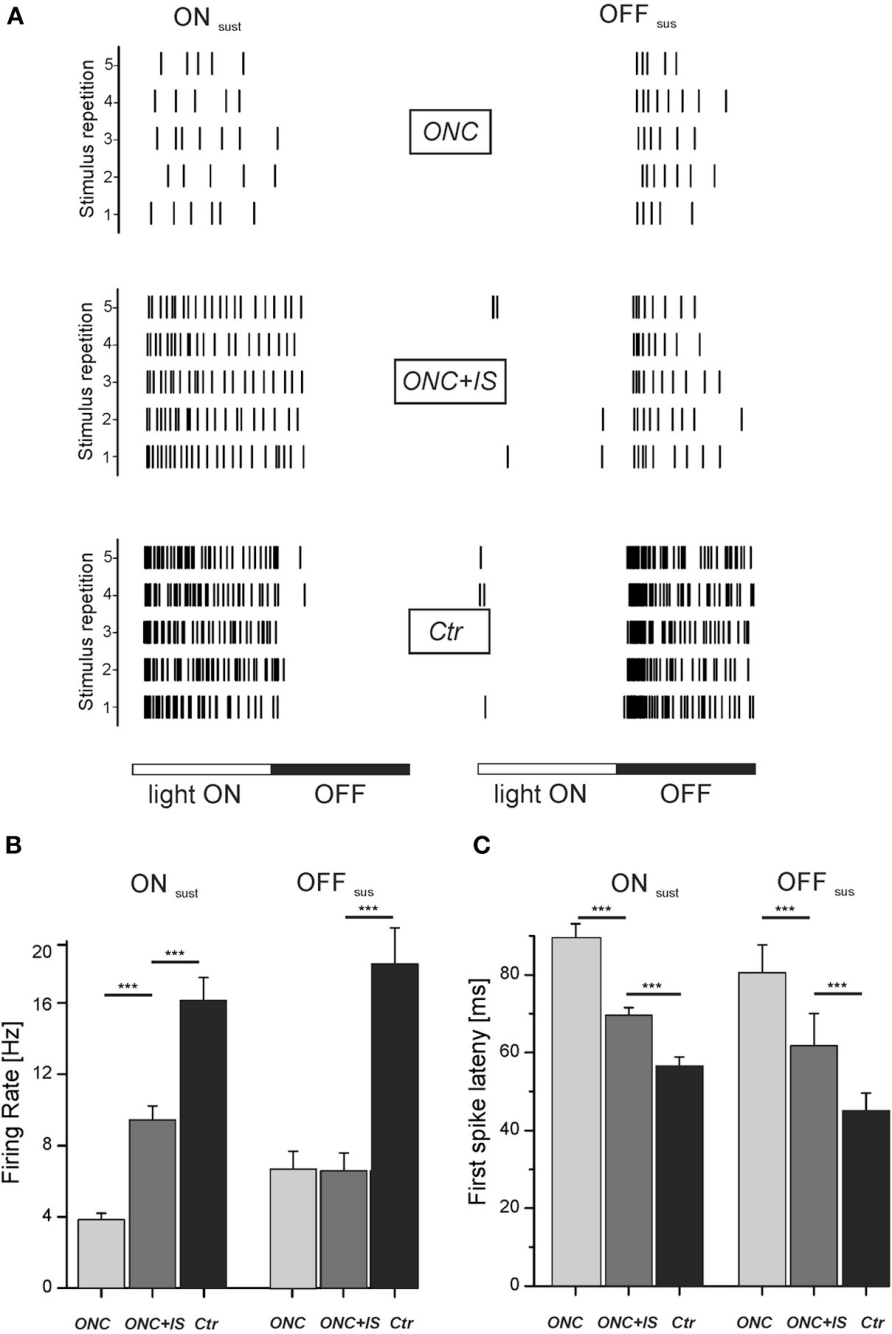
**Light-induced response properties are impaired upon ONC and partially protected by IS. (A)** Rasterplots show representative light-responses of RGC to a flash-stimulus. Left column: light-response of an ON sustained RGC of each condition. Right column: light-response of an OFF sustained RGC of each condition. Each tick represents one action potential. Each row represents one stimulus presentation. Stimulus duration is 1000 ms with each phase lasting 500 ms. **(B)** Mean firing rate of all ON_sus_ and OFF_sus_ RGCs for each condition during the corresponding stimulus phase (light ON vs. light OFF). For ON_sus_ cells, mean stimulus-induced firing rates are significantly different in ONC and ONC + IS retinae (^***^p < 0.001). Numbers of evaluated RGCs are given in the text. **(C)** Mean first-spike latency of all ON_sus_ and OFF_sus_ RGCs for each condition. Mean first-spike latency represents the occurrence of the first spike upon stimulus onset averaged over stimulus repetitions. Mean latencies of RGCs from ONC, ONC + IS and control retinae are significantly different (^***^p < 0.001). Numbers of evaluated RGCs are given in the text.

To evoke activity in the majority of RGCs in a retinal portion, we flashed large stimuli onto the retina. ON RGCs were identified based on the response to stimulus onset and the lack of spiking during stimulus offset (Figure 4A). Conversely, OFF RGCs were identified based on the response to stimulus offset and the lack of spiking during stimulus onset. Before stimulus onset retinae were adapted to the background light level for 10 min. For each recorded RGC we calculated the first spike latency and the stimulus induced firing rate (i.e., the mean number of spikes per stimulus phase divided by the duration of stimulus phase). We identified only a low number of transient RGCs for both ON and OFF cells and therefore excluded these cells from the following statistical comparison.

In the ONC condition the mean firing rate of ON_sus_ cells during light onset was 3.8 ± 0.4 Hz (*n* = 43). By additional IS the mean firing of ON_sus_ RGCs increased significantly (*p* < 0.001) to 9.4 ± 0.8 Hz (*n* = 110) (Figure 4B). This value was, however, still significantly (*p* < 0.001) lower than the mean firing rate of ON_sus_ RGCs in control retinae (16.1 ± 1.3 Hz, *n* = 135). Similarly, ONC also significantly decreased the firing in OFF_sus_ cells (6.7 ± 1.1 Hz (*n* = 14) compared to 18 ± 2 Hz (*n* = 54) in naive retinae. For OFF_sus_ cells IS did not prevented this reduction (6.5 ± 1 Hz, *n* = 14). However, this observation could be due to the relatively low number of identified OFF cells. Secondly, we note that the median firing rates were different in the two conditions: it was smaller in ONC compared to ONC + IS.

We next compared the latency of the first spike to stimulus onset. Therefore the first spike latency over 30 stimulus repetitions was averaged for each RGC. For ON_sus_ RGCs in the ONC condition the mean first spike latency was 90 ± 4 ms (*n* = 43). This value was significantly lower (*p* < 0.001) for ON_sus_ RGCs in the ONC + IS condition (70 ± 2 ms, *n* = 110) (Figure 4C). The mean first spike latency of ON_sus_ RGCs in control retinae (57 ± 2 ms, *n* = 135) was, however, significantly (*p* < 0.001) shorter than all other average values. The same trend was seen for the OFF_sus_ RGCs. The longest latency (80 ± 3 ms, *n* = 14) was measured for RGCs in ONC retinae followed by the significantly different mean latency of 61 ± 8 ms for RGCs in the ONC + IS retinae. The control OFF_sus_ RGCs (*n* = 54) displayed a mean latency of 44 ± 4 ms only.

In summary, RGCs of both investigated types showed a significant increase of response latency after ONC. This increase was significantly reduced by IS. Similarly, the maintained activity during light presentation was significantly increased in ON_sus_ RGCs in ONC + IS retinae.

## Discussion

The present study characterized electrophysiological properties of RGCs in a naive, degenerative or regenerative state. The degenerative state is achieved by optic nerve cut (ONC) and the regenerative state by additional IS. The most prominent finding is that the reduced axonal conduction velocity, observed in degenerating RGCs, is rescued by additional IS. The axonal conduction velocity of RGCs in a regenerative state is as high as in naive RGCs. Furthermore, the impaired light-response activity detected upon nerve injury is also prevented by additional IS.

These data suggest that IS does not only protect injured RGCs morphologically from cell death but also preserves their functionality. We discuss how the physiological results compare to reported histological alterations and how this information could be used to develop new regenerative strategies.

### Functional changes of intraretinal axons

Focal lesions of the optic nerve induce degenerative changes in the proximal axons of RGCs. Apart from the reduction of axon number (Berkelaar et al., 1994), histological alterations may include (1) a reduction of the axon diameter (Richardson et al., 1982) or axonal deformations (Germain et al., 2007) (2) untrastructural changes including loss of mitochondria, Nissl bodies (Barron et al., 1986) and cytoskeletal breakdown (Wang et al., 2012), and (3) a preferential loss of small axons vs. large axons (Baltan et al., 2010). The reduction of the axon diameter as well as ultrastructural changes would directly affect the axonal conduction velocity of individual axons, whereas a preferential loss of small axons would affect the average conduction velocity evaluated for a population of axons.

Following classical cable theory (Hodgkin, 1954; Matsumoto and Tasaki, 1977) the conduction velocity of unmyelinated axons scales linearly with the square root of the product of axon diameter, intraaxonal conductivity and transmembrane conductivity in its excited state. Therefore, our result of reduced axonal conduction velocity after ONC (Figure 2) may be attributed to either (1) a decrease in axon diameter, (2) a decrease of maximal transmembrane resistance, or (3) a decrease of intraaxonal conductivity. Firstly, histological studies report that the axon diameter remains intact in the first week following optic nerve injury (Richardson et al., 1982). A histological evaluation of the recorded retina is not feasible, as detachment from the recording array damages the interfaced tissue. Secondly, the amplitude and the width of extracellular somatic RGC signals does not change in the three investigated conditions (data not shown), indicating that the maximal transmembrane currents—at least in the cell soma—do not change. The decrease of conduction velocity may therefore be attributed to debris accumulation in the axon which leads to a lower conductivity (Wang et al., 2012). IS seems to prevent the accumulation of debris in the intraretinal axon—an hypothesis which needs further evaluation.

The dropout of small, slow-conducting axons in the optic nerve was suggested in a recent study in glaucomatous mice (Baltan et al., 2010). A preferential loss of slow-conducting intraretinal axons cannot be confirmed by our study. If slow-conducting axons would disappear but the fast-conducting ones would survive, the average conduction velocity should increase following ONC—in contrast to our results (Figure 2). Moreover, our data show that the variance of conduction velocities (500 mm/s) is similar for degenerative, regenerative and naive conditions. We thus infer that either all axons degenerate in a similar way or that the dropout of slow axons is accompanied by a slow-down of the fast ones. We did not identify any cell-type specific change of axonal velocity as ON and OFF ganglion cells' velocities were equally affected by ONC and equally rescued by IS.

Three experimental objections regarding axonal velocity need to be addressed. Here intraretinal conduction velocity was evaluated in *ex vivo* retinae, which implies that all axons were transected immediately before the recording. We are confident that this acute injury does not affect the velocity evaluation by comparing earlier *in vivo* (Stanford, 1987) and *ex vivo* recordings (Zeck et al., 2011). In both preparations a similar velocity range (1–2 m/s) is reported. Secondly, we do not expect any dependency of the axonal conduction velocity on the retinal location. The rat retina—in contrast to the cat or primate retina—does not show an RGC density gradient which is accompanied by a slight intraretinal velocity gradient. Thirdly, we would like to point out that in the retina—in contrast to cultured neurons—the axons run straight toward the optic nerve head. Velocity evaluation is therefore not biased by curved axonal pathways, as it seems to occur in cultured neurons (Bakkum et al., 2013). In a previous study we could show that in the rabbit retina single axonal action potentials were identified without averaging and without velocity variation along the propagation path (Zeck et al., 2011).

### Functional changes of the retinal ganglion cell

Besides the axons as the primary site of degeneration the somata and dendrites of RGCs are also affected by optic nerve injuries. It is well established that RGC density decreases following optic nerve transection (Berkelaar et al., 1994). The first significant loss in the rodent retina has been reported to occur around day 6 (rat: Fischer et al., 2004, mouse: Germain et al., 2013). Furthermore, it has been reported that somata and dendrites of the surviving RGCs shrink (Thanos, 1988; Leung et al., 2011; Kalesnykas et al., 2012). We attempted to stain the RGC and their dendrites by retrograde labelling with a fluorescent dye as described in Zeck et al. (2005). However, evaluation of the full ganglion cell dendritic tree after the recording is not feasible because in the upright microscope used here the fluorescent light is strongly scattered.

Despite the cited morphological studies, little is known about the functionality of surviving RGCs in the degenerative and regenerative state. Consistent with the current study, Takao et al. report a low spontaneous activity in both ON and OFF RGCs following optic nerve cut in the cat (Takao et al., 2002) (Figure 3). Similarly, although the light response of surviving RGCs was preserved upon nerve injury, its strength decreased over time. Other studies investigated the functionality of RGC in glaucoma models, where optic nerve constriction but also high intraocular pressure affects the RGCs. Weber and Herman investigated primate RGCs (Weber and Harman, 2005) while Feng et al. recorded mouse RGCs (Feng et al., 2013) 1 month after the induction of high intraocular pressure. Both studies report a decreased visual responsiveness to light stimulation, reflected by a reduced firing rate or a smaller receptive field size. Here we observed similar effects—even though injured RGC still show a robust light response to stimulus repetition, the light-induced firing rate as well as the light-response latency is impaired upon ONC (Figure 4). These impairments may be caused by a shrinking dendritic tree, leading to less presynaptic input and therefore to weaker and delayed light responses. For a reduced number of RGCs we were able to record both, the spike latency and the axon conduction velocity. However, there is no correlation between axon conduction velocity and spike latency indicating that—within the limited number of data—somatic activity and axonal conduction velocity change independently of each other.

In rodents, the maintained activity of ON and OFF RGCs differs significantly. Maintained activity of ON cells is largely attributed to synaptic input whereas the OFF cell activity is assumed to arise from both cell-intrinsic mechanisms and synaptic input (Margolis and Detwiler, 2007; Sekirnjak et al., 2011). Our results (Figure 3) confirm the reported differences. Following ONC (1) the maintained activity of both cell classes decreases but (2) the difference between ON and OFF cells persists. This indicates that both generating mechanisms—cell-intrinsic and synaptic mechanisms—are affected by ONC.

We further note that, following ONC, the energetically expensive firing rate (Lewis et al., 2014) decreases fourfold (Figures 3, 4B). IS preserves to a certain degree the functionally important light-induced firing rate of ON RGCs. On the other hand, synaptic integration capacity decreases only by 40% (Figure 4C) following ONC. The change of this value is reduced by IS by 25%, a value comparable to the effect on the axon.

We therefore speculate that the energetically expensive spike generation mechanism is strongly affected by ONC and IS shows a certain preservation only for a subset of cells (Figure 4B). On the other hand membrane characteristics of dendrites and axons, show less degradation and can be better preserved by IS.

### Future rescue strategies

Studies on the single cell level are important to obtain a deeper understanding how to achieve clinical regenerative treatments. The current study demonstrates that surviving RGCs remain functional 5–7 days after IS treatment. This functionality may be even augmented by additional stimulation (compare Figures 3, 4) using physiological stimuli (here: light), pharmacological agents and/or electrical pulses. Regeneration treatment with BDNF (Weber and Harman, 2008) and/or employing electrical stimulation (Morimoto et al., 2002) reportedly rescues the number of surviving RGCs. Moreover, intravitreal application of CNTF and LIF or their continuous release from retinal cells after gene therapeutic viral approaches reportedly protect RGCs from cell death and transform these neurons into a robust regenerative state, enabling these neurons to regenerate axons over long distances (Leaver et al., 2006; Muller et al., 2007, 2009; Leibinger et al., 2009; Pernet et al., 2013). These effects can be further increased when animals are co-treated with cAMP analoga (Muller et al., 2007; Fischer, 2012). In addition, recent reports have demonstrated that direct modulation of signaling pathways activated by CNTF or LIF, like the PI3K/AKT/mTOR or the JAK/STAT3 signaling pathways potently induce neuroprotective and regenerative effects on RGCs (Park et al., 2008; Sun et al., 2011; Leibinger et al., 2012, 2013).

Whether potent regenerative treatments mentioned above or others maintain RGCs functional particularly after longer times after optic nerve injury needs to be investigated in the future.

## Conclusion

The results of the present study demonstrate that transformation of RGCs into a regenerative state by IS is not only neuroprotective and stimulates axon regeneration, but it also preserves electrophysiological functions in surviving neurons—a finding with potential relevance from a clinical perspective.

### Conflict of interest statement

The authors declare that the research was conducted in the absence of any commercial or financial relationships that could be construed as a potential conflict of interest.
